# Radioanatomy of the Silent Sinus Syndrome

**DOI:** 10.1002/oto2.70212

**Published:** 2026-03-03

**Authors:** Paula von der Lage, Adrian Dalbert, Michael Soyka

**Affiliations:** ^1^ Department of Otorhinolaryngology, Head and Neck Surgery University Hospital Zurich, University of Zurich Zurich Switzerland

**Keywords:** chronic maxillary atelectasis, imploding antrum syndrome, maxillary sinus hypoplasia, silent sinus syndrome

## Abstract

**Objectives:**

Silent sinus syndrome (SSS) arises from negative pressure in the maxillary sinus through occlusion of the ethmoidal infundibulum. Convexity of the surrounding bone towards the lumen and a hypoglobus can occur. It may endanger surrounding structures during endoscopic sinus surgery. This research identifies and quantifies radiological changes in inferoposterior anatomical structures associated with SSS.

**Study design:**

Retrospective case series study.

**Setting:**

Computed tomography and magnetic resonance imaging scans of 23 patients with SSS who were treated at the University Hospital of Zurich between 2013 and 2023 were evaluated.

**Methods:**

The volume of the maxillary sinus, the orbital floor and sinus rear wall convexity, the height of the tuber maxillae, and the pterygopalatine fossa dimension were measured. The affected side was quantitatively compared with the healthy side.

**Results:**

The extension of the pterygopalatine fossa and the height of the tuber maxillae were significantly larger on the affected side (mean difference = 2.354 mm, 4.99 mm; *P* < .01 for both). A stronger indentation of the back wall (mean difference = 3.763 mm; *P* < .01) and the orbital floor (mean difference = 2.268 mm; *P* < .01) was measured in SSS. In addition, the syndrome reduces the volume of the affected maxillary sinus (mean difference = 8.885 mL; *P* < .01).

**Conclusion:**

SSS causes a marked enlargement of the neighbouring inferoposterior anatomical spaces. Shrinking of the sinus' volume could explain this effect. These findings have direct clinical implications, as the surgeon might inadvertently enter these structures more easily in SSS.

Silent sinus Syndrome (SSS) is characterised by maxillary sinus atelectasis combined with indented sinus boundaries. Patients suffering from SSS complain mostly about slowly progressing facial asymmetries and sometimes about diplopia.^1^ Mainly, clinical examination reveals an enophthalmus with a hypoglobus on the affected side. In the end, a radiological examination is essential. The gold standard for radiological examination is a skull computed tomography (CT) while magnetic resonance imaging (MRI) is infrequently performed.^2^, ^3^, ^4^, ^5^ CT or MRI shows typical features of the affected sinus: maxillary sinus atelectasis with convex walls towards the antrum, an opacified maxillary sinus, depressed and thin orbital floor with an enlarged orbital cavity, a lateralized uncinate process, a blocked osteomeatal complex and a nasal septal deviation with bending towards the affected sinus.^1^, ^6^, ^7^ It is assumed that the ostium naturale closes, meaning that secretions can no longer drain out of the sinus. As a result, gases contained in the sinus are resorbed and a negative intrasinusal pressure occurs. The vacuum created inevitably leads to the retraction of the sinus walls into the maxillary cavity.^2^, ^8^, ^9^, ^10^, ^11^, ^12^

Treatment of choice is functional endoscopic sinus surgery (FESS) aiming to improve maxillary sinus drainage by means of an uncinectomy and a middle‐meatal antrostomy. The surgeons may encounter adipose tissue. This usually indicates inadvertent orbital entry.^13^ In SSS, fatty tissue could also originate from the anatomically adjacent pterygopalatine fossa, when negative pressure in the sinus brings the posterior wall anteriorly.^3^, ^14^ To date, increased retroantral adipose tissue has only been described in a few studies and patients.^15^, ^16^

Too little is understood about the enlargement of the surrounding anatomical cavities and their increased retroantral fat deposits. In particular, the inferior and posterior anatomical environments are poorly understood. This study aimed to describe anatomical features of SSS‐affected maxillary sinuses on CT and MRI scans. The focus was on the identification of radiographic characteristics of structures inferoposterior of the maxillary sinus. Size change of the tuber maxillae, pterygopalatine fossa expanse and its containing adipose tissue were measured and compared to the healthy side. In addition, maxillary sinus volume was calculated.

## Materials and Methods

### Study Design and Population

The consent of the participants was obtained through a signed general consent of the University Hospital Zurich. Patients who were treated at the University Hospital Zurich before 2016 (prior to the introduction of the general consent) and could not be contacted within a month were included in the study without signed general consent. This was explicitly authorised by the cantonal ethics commission Zurich. This research was permitted by the Swiss ethic committee of the canton Zurich (No. KEK 2022‐01784). The project was carried out in compliance with the project plan, the current version of the Helsinki Declaration and Swiss legislation.

This retrospective case series study included patients from the University Hospital Zurich who were treated for SSS in the Department of Otorhinolaryngology, Head and Neck Surgery or the Department of Ophthalmology between 2013 and 2023. SSS was defined according to Stryjewska‐Makuch et al as a complete opacity of the maxillary sinus with an accompanying convexity of one bony boundary of the sinus.^6^ Patients with bilateral SSS, previous surgery in the sinonasal area or existence of an endonasal neoplasia were excluded. Suitable patients were recruited from the internal hospital information system (KISIM) and the ENT Statistics software using the keywords SSS, imploding antrum, and chronic maxillary atelectasis. These patients received either a CT or an MRI scan of the skull and paranasal sinuses, which were analysed in a standardised manner. All patient data and images were taken out from KISIM and from the picture archiving and communication system (PACS). A total of 30 patients met the requirements and could be included. Of these, 7 patients had an MRI scan and 23 received a CT image for diagnostic purposes.

### Scan Characteristics

Overall, 23 CT scans were analysed in all 3 projections (coronal, sagittal, and axial). The measurements of the orbital cavity were taken in the soft tissue window for a better contrast. All other structures were measured in the bone window. To guarantee standardised implementation, each central plane of maxillary sinus expansion from all scans in the 3 dimensions were measured. The central plane was determined by adding 1 to the sum of CT slices of sinus expansion dividing by 2. Decimal values have always been rounded. The following types of acquisition were used in the 7 MRI images: T1 spin echo (SE), 3D Fluid attenuated inversion recovery (flair), 3D flair multiplanar reconstruction (MPR), T2 turbo spin echo (TSE), fast spoiled gradient‐echo (FSPGR) natively or with gadolinium.

### Scan Analysis

**Figure 1:**
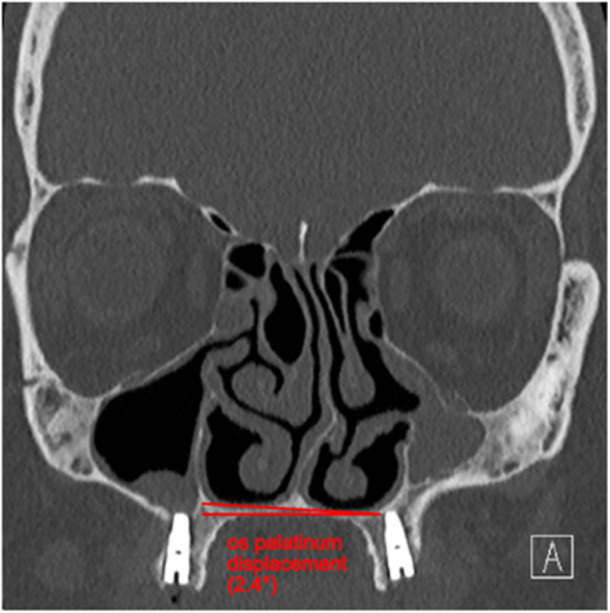
Measurement of os palatinum displacement. The angle of 2.4° describes the lowering of the affected sinus side.

**Figure 2:**
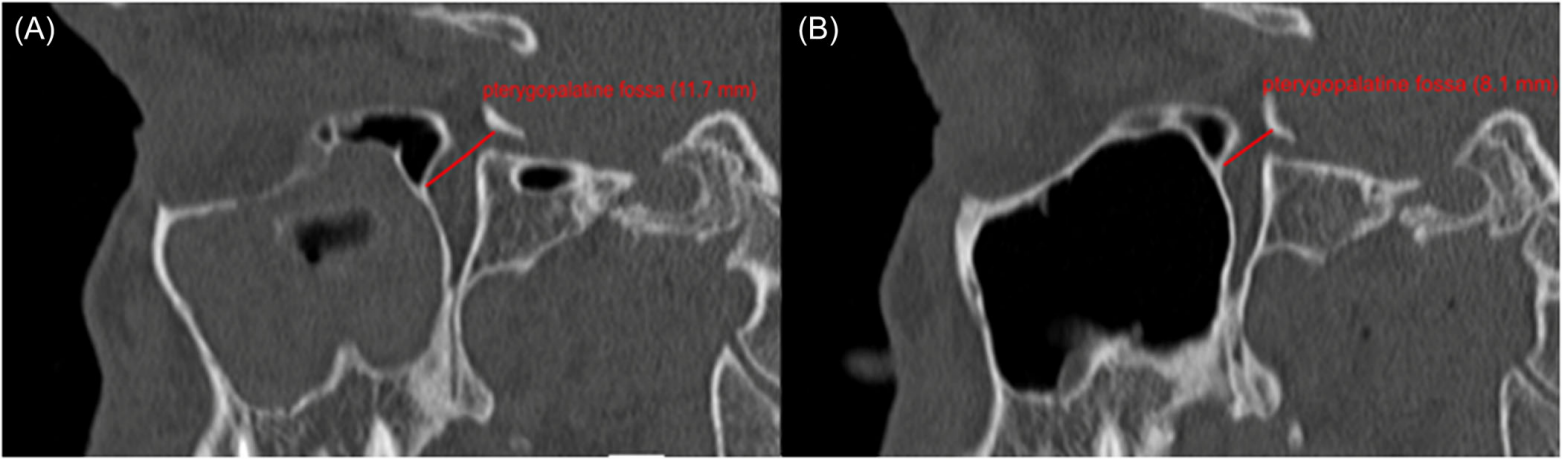
Sagittal skull CT scan of a patient suffering from SSS, (A) pterygopalatine fossa width of the SSS‐affected side (right maxillary sinus), (B) pterygopalatine fossa width of the healthy side (left maxillary sinus). CT, computed tomography; SSS, silent sinus syndrome.

**Figure 3:**
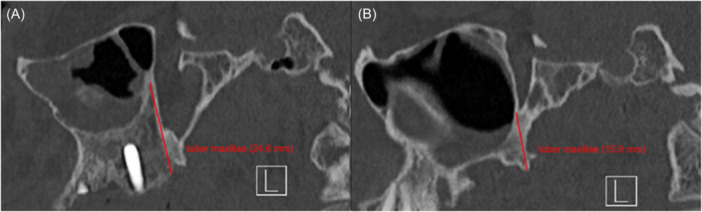
Sagittal skull CT scan of a patient suffering from SSS, (A) Tuber maxillae height of the SSS‐affected side (right maxillary sinus), (B) tuber maxillae height of the healthy side (left maxillary sinus). CT, computed tomography; SSS, silent sinus syndrome.

The scans were analysed in the 3 programs synedra View (version 23.0.0.2), DeepUnity Diagnost (version 1.1.1.1), and 3D Slicer (version 5.4.0.). The volume of the maxillary sinus was calculated using digital imaging and communications in medicine (DICOM) files in the 3D Slicer. The width of the pterygopalatine fossa and the height of the maxillary tuberosity were measured in the sagittal section at the level of the foramen rotundum (Figures 2 and 3). Sagittal and coronal total expansion of the maxillary sinus and its rear wall convexity towards the sinus were recorded in the exact central axial plane of the sinus' full expansion. The measurement of the rear wall convexity was defined as the distance between the rear wall and a connecting line between the zygomatic recess and the medial maxillary sinus wall. A positive value was measured when the connecting line was located posterior of the sinus' back wall. The higher the value the greater the indentation of the rear wall was. The back wall thickness was measured in the axial projection at the thickest area. Thickness and lowering of orbital floor were both measured in the exact central plane of the coronal projection of the sinus' whole expansion. The orbit was sized by quantifying the distance from the roof of the orbit to the upper edge of the eye bulb and the distance from the lower edge of the eye bulb to the orbital floor. Nasal septal deviation was measured as an angular deviation in the axial and coronal plane. The frontal sinus was measured on both sides with complete representation above the orbit on the same scan. Os palatinum displacement was determined by measuring the angel between a horizontal connecting line and the os palatinum. For details, refer to the Figure 1.

### Statistics

R programming language (version 4.3.2) with the development environment of RStudios (2023.12.0) for programming and graphical visualisation served for all statistical analysis. Normal distribution was investigated graphically and confirmed by the the Kolmogorov‐Smirnov test. A paired *t‐*test was used to statistically compare the healthy with the affected side for Gaussian distributed data. Statistical comparison of nonnormally distributed data was carried out using the Mann‐Whitney‐*U*‐test. The correlations of individual measurements were analysed using Pearson correlation and Spearman rank correlation. Statistical significance was defined as *P* < .05.

## Results

Overall, 30 patients (16 women [53%], 14 men [47%]) were included in this study. The average age of all included patients was 44.9 years with a mean age of 43.8 years in the female cohort of 45.6 years in the male. Among the 30 patients analysed, 23 patients received a CT scan of the skull and 7 patients received a skull MRI as part of the SSS diagnostic workup. In 18 of the 30 patients the SSS was localised on the right side (60%).

**Figure 4:**
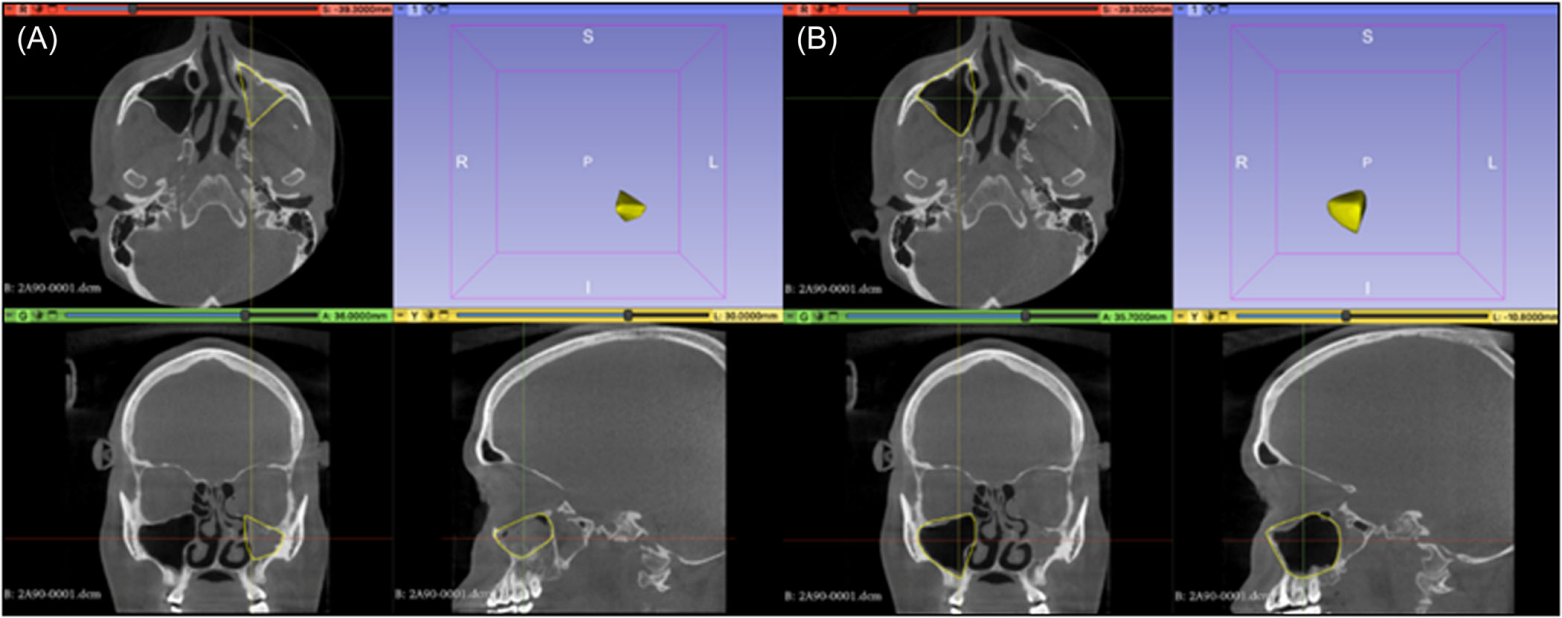
Volume measurement in the 3D Slicer. (A) SSS affected maxillary sinus, (B) healthy maxillary sinus. I, inferior; L, left; P, posterior; R, right; S, superior. SSS, silent sinus syndrome.

**Figure 5:**
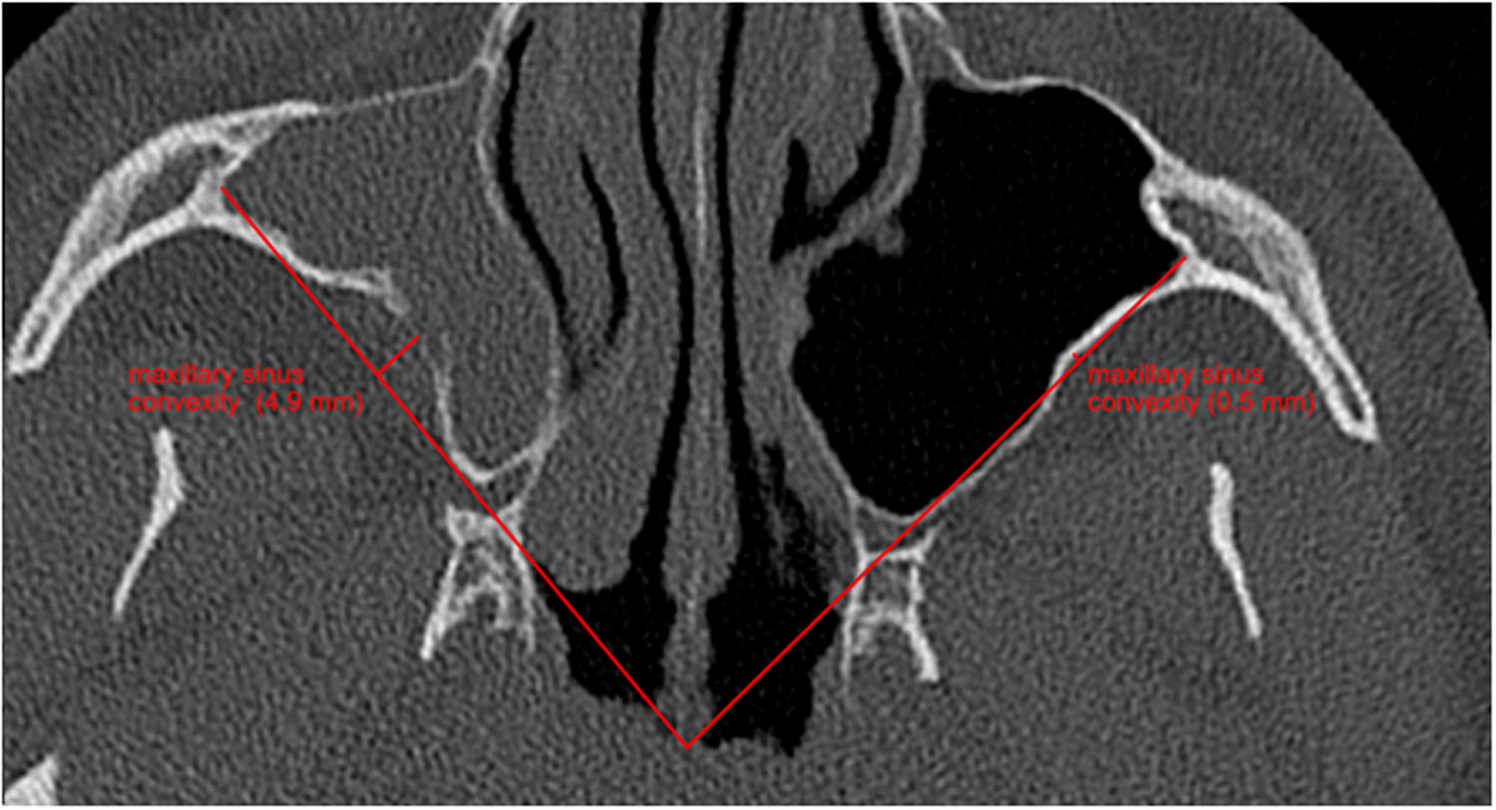
Measurement of the posterior wall convexity of the maxillary sinus in the axial CT scan with a left healthy sinus and an SSS‐affected sinus on the right side. CT, computed tomography; SSS, silent sinus syndrome.

In all 30 patients, a completely opacified maxillary sinus was described on the affected side. 26 of 30 patients showed a deviation of the nasal septum in direction of the diseased maxillary sinus (87%). The pterygopalatine fossa was significantly widened compared to the healthy side (mean difference 2.354 mm; *P* < .01). There was also a significant increase in the tuber maxillae height on the SSS side compared to the unaffected sinus (mean difference = 4.99 mm; *P* < .01). Pterygopalatine fossa and tuber maxillae measurements are shown in Figures 2 and 3. The average volume of the affected and the healthy sinus measured 10.057 and 18.942 mL, respectively (Figure 4). The volume of the affected maxillary sinus was thus significantly smaller (mean difference 8.885 mL; *P* < .01). The posterior wall of the maxillary sinus showed a significant greater convexity towards the sinus on the affected side (mean difference = 3.763 mm; *P* < .01). Convexities of both sides are represented in Figure 5. Both sagittal (mean difference = 5.53 mm; *P* < .01) and coronary length (mean difference = 6.23 mm; *P* < .01) confirmed a diameter reduction of the SSS sinus. For orbital floor displacement, distance between bulbus oculi and orbital floor was measured and calculated. Nevertheless, this measurement method would have missed the position change of the orbital floor if the bulbus occuli was simultaneously lowered. Therefore, the effective lowering of the orbital floor was determined in a second indirect method. Distance from orbital roof to bulbus oculi was summed with the distance from bulbus oculi lower edge to orbital floor. This calculated value is defined in the current study as orbital floor lowering. Both measurement methods showed a significant lowering of the orbital floor on the affected side. No significant difference in the ventilation of the respective equilateral frontal sinus could be shown (*P* = .201). The detailed measurements and respective *P*‐values are summarised in Table 1.

**Table 1:** Values of all Measurement and Comparison of Healthy Maxillary Sinus Versus SSS

	Healthy maxillary sinus			SSS maxillary sinus			Healthy vs. SSS
	Mean	± SD	Median	Mean	± SD	Median	*P*‐values
Posterior wall maxillary sinus (mm)							
Thickness	1.231	± 0.321	1.1	1.527	± 0.819	1.3	.128
Convexity	−7.503	± 3.228	−7.7	−3.74	± 3.85	−4.65	**<.01**
Maxillary sinus diameter (mm)							
Sagittal	36.93	± 4.427	36.8	31.4	± 5.814	31.85	**<.01**
Coronary	25.05	± 4.245	25.4	18.82	± 5.612	19.05	**<.01**
Maxillary sinus volume (mL)	18.942	± 7.992	18.043	10.057	± 6.222	9.691	**<.01**
Tuber maxilla height (mm)	12.99	± 4.114	12.80	17.98	± 5.09	15.40	**<.01**
Pterygopalatine fossa width (mm)	8.346	± 1.663	8.2	10.7	± 2.444	10.9	**<.01**
Frontal sinus size (mm)	26.11	± 10.628	27.9	22.52	± 13.095	23.7	.201
Orbital floor (mm)							
Thickness	1.154	± 0.424	1.1	1.054	± 0.323	1.1	.476
Convexity	0.066	± 2.181	0.085	−2.202	± 2.608	−1.7	**<.01**
Lowering	14.81	± 2.282	15.15	16.95	± 2.793	16.8	**.003**
Orbita (mm)							
Distance orbital roof to bulbus oculi	6.948	± 1.603	6.7	7.419	± 1.627	7.42	.276
Distance orbital floor to bulbus oculi	7.861	± 1.74	8.025	9.527	± 2	9.4	**.001**
Nasal septum deviation (°)							
Axial				5.517	± 5.879	5.75	
Coronary				6.231	± 7.564	7.3	
Os palatinum (°)				−0.396	± 3.316	−1.1	

The significant *P*‐values are highlighted in bold.

**Figure 6:**
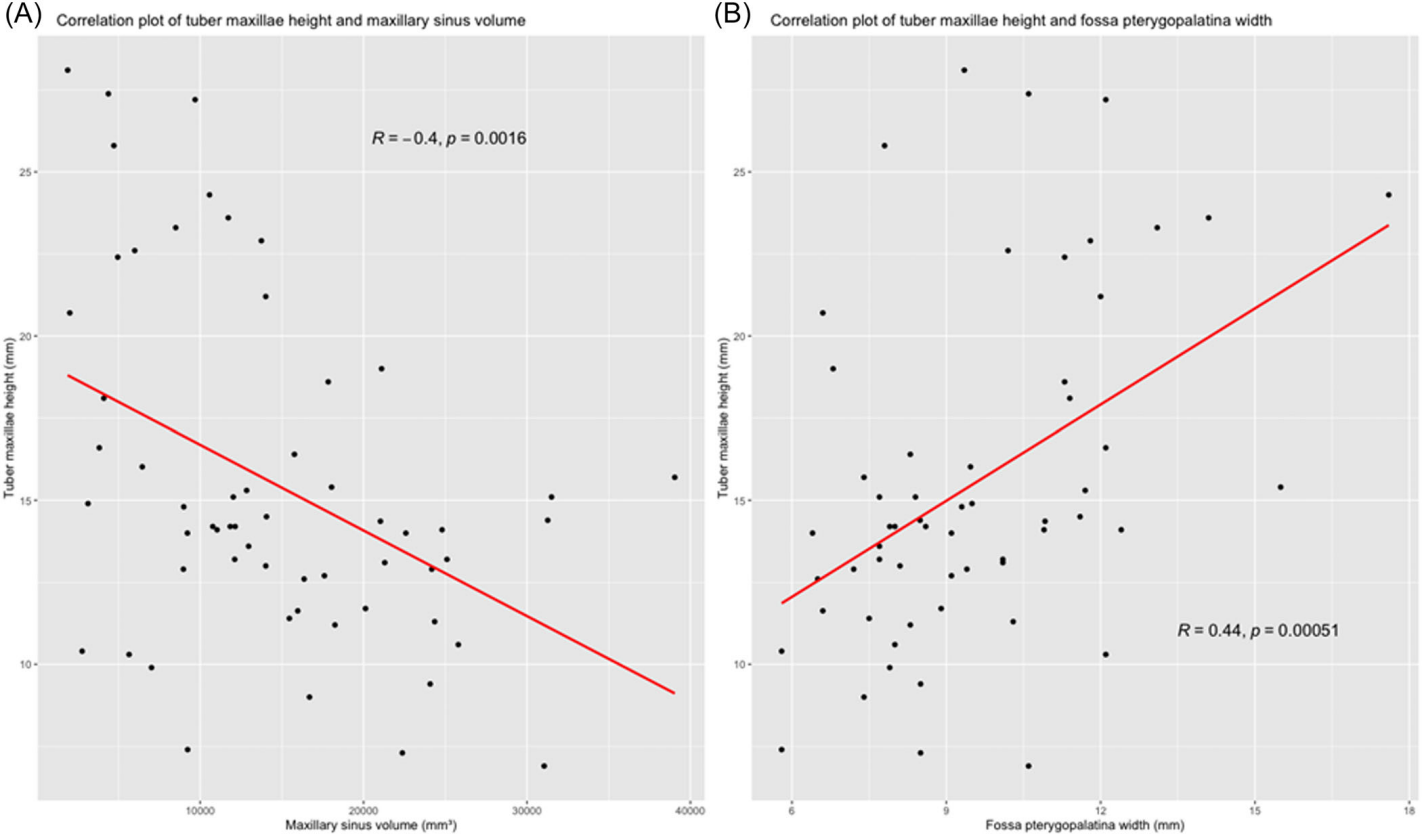
Correlation plots, (A) correlation of tuber maxillae height with maxillary sinus volume, (B) correlation of tuber maxillae height and pterygopalatine fossa width.

The height of the tuber maxillae correlated negatively with the volume of the sinus (rs = −0.404, *P* = .02). No correlation could be found between the volume of the sinus and the width of the pterygopalatine fossa (rs = −0.187, *P* = .158). However, the height of the tuber maxillae correlated with the width of the pterygopalatine fossa (rs = 0.442, *P* < .01) (Figure 6A). Moreover, a positive correlation was described between both the sagittal length (*r* = 0.732, *P* < .01) and the coronal length (*r* = 0.856, *P* < .01) and the maxillary sinus volume (Figure 6B). No correlation was found between the inclination of the os palatinum and the deviation of the septum (rs = −0.161, *P* = .404) or the maxillary sinus volume (rs = 0.176, *P* = .36).

## Discussion

This retrospective study investigated radiological properties of an SSS‐affected maxillary sinus. We showed that the tuber maxillae enlarges and the pterygopalatine fossa widens significantly in affected maxillary sinuses. In addition, we could measure the already previously described reduced diameter and the associated volume reduction of the affected maxillary sinus in relation to the healthy side. The enlargement of the tuber maxillae correlates positively with the extension of the pterygopalatine fossa and correlates negatively with the maxillary sinus volume.

The current literature, contains primarily case reports. Deeper knowledge about this little researched disease is still missing. Our aim was to gain a better understanding of the anatomical changes and conditions of SSS and its consequences for surgical therapy.

Most commonly, SSS is clinically described as an enopthalmus and hypoglobus on the affected side. In literature, the term SSS first appeared in 1994. The syndrome was then defined as the 2 above‐mentioned clinical manifestations in absence of sinonasal symptoms, trauma, or surgery.^17^, ^18^ However, already earlier in 1964 patients suffering from SSS similar symptoms have been described.^19^ The typical clinical presentation with ocular asymmetry with a one‐sided sunken eye and superior sulcus deepening results from the convexed orbital floor towards sinus antrum. Moreover, the reduced volume of the maxillary sinus explains the enopthalmus and hyopglobus.^20^, ^21^

Congruent with current literature, we were able to detect a reduced sinus volume and an inward bending of the orbital floor.^21^, ^22^ In our measurements, there were no significant thickness changes of the sinus' rear wall or the orbital floor, compared to the healthy sinus. This points to a missing chronic inflammatory process in SSS. Both a thickening as well as a thinning of the orbital floor have previously been described in the literature.^23^ In our investigations, both variants were recognized. An explanation for the thinning of the bone boundary could be a mild inflammatory reaction in the sinus and a reduced activity of osteoblasts due to subathmospheric pressure and thus promotion of osteolysis, leading to thinning or even fracturing of the orbital floor. This could lead to a pathological communication between the sinus and the pterygopalatine fossa or the orbit.^4^, ^24^ With demineralised bony boundaries, there is a greater risk of unwanted penetration into neighbouring anatomy in FESS.

The underlying mechanisms leading to SSS are still controversial. Baujat et al proposed a mechanical theory for the development of negative pressure. They postulated based on a case report that the masticatory movement through contraction of the lateral pterygoid muscle and temporalis muscle results in negative pressure in the pterygopalatine fossa and thus displaces orbital fat into the fossa. The posterolateral wall then bends into the maxillary sinus due to the negative pressure generated.^25^ The second hypothesis assumes an obstructed outflow of the maxillary sinus and as a consequence, the development of negative pressure within the maxillary sinus. To date, these two are the two relevant pathophysiological hypotheses in SSS.^26^

We were also able to show that almost all patients develop a nasal septal deviation towards the smaller sinus. This characteristic was described previously.^3^ We were also interested in the size change and pneumatisation of the frontal sinus. However, we could not detect any change compared to the healthy side. In addition, the inclination angel of the os palatinum revealed also no difference between the affected and the healthy side.

The measurement of the tuber maxillae height was carried out for the first time in literature. We measured significant tuber maxillae length increase of the diseased maxillary sinus. This new insight explains a considerable increase of distance between the molar roots and the jaw cavity. Furthermore, the height of the tuber maxillae correlates with the width of the pterygopalatine fossa and correlates negatively with the reduction of the equilateral maxillary sinus. It is intriguing to state that SSS could also have an influence on the upper denture via the enlargement of the tuber maxillae. Since the upper jaw teeth with their roots are anchored in the maxilla, an anatomical shift of the tuber maxillae could have a significant influence on the dental anatomy.^27^ So far, only few cases have been described in which SSS has been diagnosed by dental panoramic radiography with a shaded maxillary sinus.^28^ However, to our knowledge there are no published cases of dental problems in association with SSS.

Rarely, the term imploding antrum syndrome is used in literature instead of SSS.^7^ Besides, the term chronic maxillary atelectasis (CMA) appeared in 1992 in literature and has to be distinguished from SSS. CMA is described as a decrease in the volume of the maxillary sinus with a subsequent convexity of the bony sinus surrounding. Whereas in SSS the focus lies on enopthalmus and hypoglobus without sinonasal symptoms. CMA is graded into three clinical levels. Grade I and grade II describe the anatomical properties and changes of CMA. Grade III is characterized by the additional presence of clinical deformities such as facial asymmetries due to a hypoglobus or enopthalmus. SSS and clinical stage III CMA are therefore considered to be part of the same disease spectrum. In SSS, sinonasal symptoms are absent compared to CMA. Until now, scientists still disagree on nomenclature.^24^ According to de Dorlodot et al.'s definition, the 30 patients studied in our study are classified as followed: 21 patients with CMA I/II, 4 patients with CMA III and 5 patients with CMA III or SSS. Nevertheless, it remains undisputed, that the same surgical procedure is performed regardless of the used classification system.^24^, ^29^, ^30^, ^31^ FESS is the standard treatment for SSS. This procedure can lead to complete regress of the enopthalmus and hypoglobus within a few weeks.^9^, ^16^ In some patients, an orbital floor reconstruction with an allogenic or autologous graft is carried out to stabilise the thin wall between the orbit and the maxillary sinus. Orbital reconstruction is performed either during FESS or in a separate procedure.^1^, ^32^

A limitation of this study is the exclusion of bilateral SSS in order to compare the healthy side with the SSS‐affected side in the same patient. Although, it was long assumed that the SSS appears only unilaterally, a few cases of bilateral SSS have been described in recent years.^24^, ^33^ In our retrospective database analysis, we could identify one case of bilateral SSS.^1^ A further limitation is the exclusion of patients with partially aerated maxillary sinus. It is conceivable that SSS cases with only partially opacified maxillary sinus exist.^21^ One of the strengths of the study is the formulation of a standardised procedure for the measurement of the CT scans. This ensured the comparability of the results between different patients.

Knowledge of the inferior and posterior enlarged structures are important indicators for potential risks in the surgical treatment of an affected maxillary sinus. The current study provided further insights into the poorly understood anatomical changes in SSS.

Exact anatomical knowledge is of great importance for a safe surgical outcome. SSS causes an extensive reduction of the maxillary sinus size and volume due to negative pressure. Specifically, the bony walls bend into the antrum, the pterygopalatine fossa dilates, and the tuber maxillae prolongates. Awareness of this changed anatomical environment is essential during surgical management of SSS.

## Author Contributions

**Paula von der Lage**: Conceptualisation, data collection, statistics, visualization, formal analysis, writing original draft; **Adrian Dalbert:** Elaborated reviewing of the results and the original draft; **Michael Soyka:** Investigation, project administration, conceptualisation, study design, methodology, supervision, validation, reviewing and editing original draft.

## Disclosures

### Competing interests

None.

### Funding source

This research was funded by Theodor und Ida Herzog‐Egli‐Stiftung, Switzerland.
